# Clustering patterns of obesity-related multiple lifestyle behaviours and their associations with overweight and family environments: a cross-sectional study in Japanese preschool children

**DOI:** 10.1136/bmjopen-2016-012773

**Published:** 2016-11-04

**Authors:** Etsuko Watanabe, Jung Su Lee, Katsumi Mori, Kiyoshi Kawakubo

**Affiliations:** 1 Department of Health Promotion Science, School of Public Health, Graduate School of Medicine, The University of Tokyo, Tokyo, Japan; 2 Department of Food Science and Nutrition, Kyoritsu Women's University, Tokyo, Japan

**Keywords:** EPIDEMIOLOGY, PUBLIC HEALTH

## Abstract

**Objectives:**

The purpose of this study is (1) to identify obesity-related lifestyle behaviour patterns of diet, physical activity, sedentary and sleep behaviours in preschool children, (2) to examine the association between identified behaviour clusters and overweight/obesity and (3) to investigate differences in children's family environments according to clusters.

**Design setting and participants:**

A cross-sectional study on 2114 preschool children aged 3–6 years who attended childcare facilities (24 nursery schools and 10 kindergartens) in Tsuruoka city, Japan in April 2003 was conducted.

**Main outcome measures:**

Children's principal caregivers completed a questionnaire on children's lifestyle behaviours (dinner timing, outside playtime, screen time and night-time sleep duration), family environment (family members, maternal employment, mealtime regularity and parents' habitual exercise and screen time) and measurements of weight and height. Cluster analysis was performed using children's 4 lifestyle behaviours based on those non-missing values (n=1545). The χ^2^ tests and analysis of variance (ANOVA) estimated cluster differences in overweight/obesity and family environments.

**Results:**

6 clusters were identified. Children's overweight/obesity varied across clusters (p=0.007). The cluster with the most screen time, shorter night-time sleep duration, average dinner timing and outside playtime had the highest overweight/obesity prevalence (15.1%), while the cluster with the least screen time, the longest sleep duration, the earliest dinner timing and average outside playtime had the lowest prevalence (4.0%). Family environments regarding mealtime regularity and both parents' screen time also significantly varied across clusters. The cluster having the highest overweight/obesity prevalence had the highest proportion of irregular mealtimes and the most screen time for both parents.

**Conclusions:**

This study suggests that public health approaches to prevent children's overweight/obesity should focus on decreasing screen time and increasing night-time sleep duration. To shape those behaviours, regular mealtimes and decreasing parents' screen time within family environments need to be targeted among family members.

**Strengths and limitations of this study**

- Preschool children's obesity-related lifestyle behaviour patterns including diet, physical activity, sedentary and sleep behaviours were identified using cluster analysis.
- The study population included all preschool children (3–6 years) who attended childcare facilities in a city with more than 100 000 population.
- This study was a cross-sectional design; measurements were based on the principal caregivers' reports and did not include socioeconomic status variables.
- Studies on clarifying the association with socioeconomic status in various communities are needed.

## Introduction

Multiple daily lifestyle behaviours including diet, physical activity, sedentary and sleep habits affect body weight status.^1–10^ Increased body weight in childhood influences several chronic diseases such as coronary heart disease, diabetes and metabolic syndrome in childhood^11^ and adulthood.^12^ High energy intake, late eating at night and excessive television (TV) viewing are associated with increased risk of overweight,^1–3^ ^6^ ^7^ while a high level of physical activity and long sleep duration have been shown to be protective measures against overweight.^3–5^ ^8–10^ These lifestyle behaviours are shaped from early childhood, and adopted lifestyle behaviours carry over into adulthood.^13^ ^14^ Hence, the development of healthy lifestyle behaviours starting from early childhood should be encouraged to achieve or maintain a healthy body weight status.

Various weight-related behaviours are related to each other, and lifestyle behaviour patterns clustered around habitual behaviours, rather than individual behaviours, are considered to be related to body weight status. It is therefore important to examine weight-related lifestyle behaviour patterns combined with individual behaviours. Several studies have examined clustering patterns of multiple lifestyle behaviours in children and adolescents.^15–21^ Most of the studies have focused on diet, physical activity and/or sedentary behaviours as weight-related behaviours. However, sleep behaviour is one of the habits related to risk of overweight in children.^5^ ^10^ Except for studies in European and Australian school-age children,^16–18^ no other studies were identified that included sleep habits. To promote healthy lifestyle behaviours during childhood, it is necessary to identify comprehensive lifestyle behaviour patterns, including sleeping habits as well as diet, physical activity and sedentary behaviours.

Children's lifestyle behaviours are affected by family environments, especially among young children. Some studies considering family environments have examined the influence of family members who live with children on those children's behaviours.^22–24^ These studies found that children with siblings were more physically active than an only child,^24^ children with one parent or a working mother spent more time watching TV^23^ ^24^ and those with a working mother also had increased high-energy drink consumption and short sleep duration.^22^ ^23^ Other studies have examined the influence of parents' habitual behaviours on children's behaviours.^25–27^ There is evidence that children with more active parents were more physically active,^25^ and children with parents watching excessive TV also spent more time watching TV.^26^ ^27^ These studies examined how the behaviours of family members living with children influenced the children's individual behaviour. However, those family environments may influence children's lifestyle behaviour patterns. Thus, it is important to assess associations of children's lifestyle behaviour patterns with both aspects of family environments.

The purpose of this study is (1) to identify lifestyle behaviour patterns of diet, physical activity, sedentary and sleep behaviours in preschool children, (2) to examine the association between identified behaviour clusters and overweight/obesity and (3) to investigate differences in children's family environments according to clusters.

## Methods

### Study design and population

This cross-sectional study was conducted in childcare facilities including nursery schools and kindergartens in April 2003. Most preschool children aged 3 and older attend such facilities in Japan. The study population included all preschool children aged 3–6 years who attended childcare facilities (24 nursery schools and 10 kindergartens) in Tsuruoka city, located in northeast Japan and their principal caregivers.

A self-administered questionnaire was delivered to each child's principal caregiver, which was returned to the child's facility after completion of the questionnaire at home. Only questionnaires in which the principal caregivers provided consent for study participation and were anonymously returned were included.

### Measures

#### Children's lifestyle behaviours

Dinner timing was used as an indicator of dietary behaviours since a significant association between late night eating and higher body mass index (BMI) has been observed in adults.^6^ Dinner timing was recorded as the usual time of eating dinner. Outside playtime and screen time were included as indicators of being physically active or inactive. Outside playtime was recorded as hours and minutes usually spent playing outside. Screen time was recorded as hours and minutes usually spent watching TV and videos and playing electronic games. Night-time sleep duration as an indicator of sleep habit was assessed by recording usual bedtime and wake-up time. Night-time sleep duration was calculated as the time elapsed in hours between bedtime and wake-up time. These behaviours for a usual weekday and weekend day were assessed separately and calculated as the mean time per day by summing up weekdays and weekend days and then dividing by seven.

#### Family environments

To examine the influence of family environments living with children on children's lifestyle behaviour pattern, parents were referred to those who live with children, regardless of whether they are biological parents or not. Principal caregivers were referred to parents or grandparents who live with and take care of children.

*Family members living with children*: Parental status was separated into two parents or one parent. Presence of siblings was categorised according to whether children lived with at least one sibling. Presence of grandparents was also categorised according to whether children lived with at least one grandparent. Maternal employment status was categorised as unemployed or employed (full-time, part-time and self-employed).

*Habitual family and parents' behaviours*: Meal regularity was divided according to whether a family has meals at regular times or irregular times. Parents' habitual exercise was assessed by asking each parent to report the frequency (days/week) and duration (minutes/day) of sports or exercise. Their responses were categorised as meeting the physical activity recommendation (150 min/week).^28^ Parents' screen time was assessed by asking each parent to record the hours and minutes usually spent watching TV and videos and playing electronic games. Screen time was calculated as the mean time per day by summing weekdays and weekend days and dividing by seven and categorised among the respective parents as <2, 2–3, or ≥4 hours/day.

#### Children's anthropometric measurements

Children's body weight (kg) and height (cm) were measured using standard methods (in light clothing and without shoes) at each facility before distributing the questionnaire, as a part of a periodic health examination. The measurements were recorded in health handbooks and given to the principal caregivers. The principal caregivers filled out the questionnaire by referring to the handbook. BMI was calculated as body weight divided by height squared (kg/m^2^). Children were classified as non-overweight or overweight (including obese) according to sex-specific and age-specific BMI cut-off points of the International Obesity Task Force,^29^ which is internationally accepted and has been used in previous childhood obesity research conducted in many countries such as Europe,^1–4^ ^16^ ^20^ ^30^ ^31^ Australia^15^ ^17^ and Japan.^32^

#### Participant characteristics

Participant characteristics included children's sex and age and parents' age, weight and height. Parents' self-reported weight and height were used to calculate their BMI, and parents' overweight (including obese) was defined as BMI≥25 kg/m^2^.^33^

### Statistical analysis

All statistical analyses were conducted using SAS V.9.3 (SAS Institute, Cary, North Carolina, USA). Cluster analysis (SAS FASTCLUS) was performed to identify subgroups with similar obesity-related lifestyle behaviours according to dinner timing, outside playtime, screen time and night-time sleep duration. Boys and girls were combined for analyses to identify representative lifestyle behaviour patterns in preschool-aged children. Variables used to assess the four behaviours were standardised (z-scores) before clustering in order to avoid the influence of variables with substantially different ranges. Cluster analysis included children who had no missing values for the behaviours and was conducted by partitioning data into different clusters (3–7) by Euclidean distances between observations.^34^ Cluster solutions are sensitive to the initial cluster centres. Therefore, in order to find optimal specifications for initial cluster centres, 1000 iterations of each cluster procedure using randomly generated initial group centres were conducted. The solution with the largest overall r^2^ value which represents relative heterogeneity between clusters compared with heterogeneity within clusters was identified. To examine the stability of the cluster solutions, the total sample was randomly divided into two subsamples in which the clustering procedure was repeated. Cohen's κ coefficient of the cluster solutions of both subsamples with that of the total sample was calculated (κ=0.92 and 0.93 for this final cluster solution). The final cluster solution was determined according to large values of the pseudo-*F* index and high interpretability and stability of cluster patterns.^15^ ^16^ ^18–21^ ^34^ ^35^

The mean values of the four lifestyle behaviours were compared across clusters using analysis of variance (ANOVA). Participant characteristics, children's weight status and family environments as variables were compared by using χ^2^ tests for frequency measures and ANOVA for continuous variables. Two-sided p values <0.05 were considered as statistically significant. The significance level for these analyses was adjusted using the Holm's method^36^ for addressing problems of multiple testing.

## Results

### Study participants

Surveyed target participant was 2114 children who attended childcare facilities in the city, and 1867 (88.3%) returned a completed questionnaire. Of these, 322 children were excluded due to missing analytic behaviour values. The final sample included 1545 (73.1%) children (825 boys and 720 girls) and the mean age was 4.2 (SD 0.9) years.

Comparing included and excluded children's characteristics, there were no statistically significant difference by children's sex (53.4% and 51.5% boys, p=0.446), age (mean 4.2 and 4.2 years, p=0.841), overweight (8.6% and 10.6%, p=0.213), or mothers' age (mean 33.5 and 33.3 years, p=0.446) and BMI (mean 21.1 and 21.2 kg/m^2^, p=0.622); whereas, fathers' age (mean 36.1 and 35.4 years, p=0.031) was older and father's BMI (mean 23.3 and 23.0 kg/m^2^, p=0.036) was larger in included children.

### Cluster patterns of lifestyle behaviours

**Figure 1:**
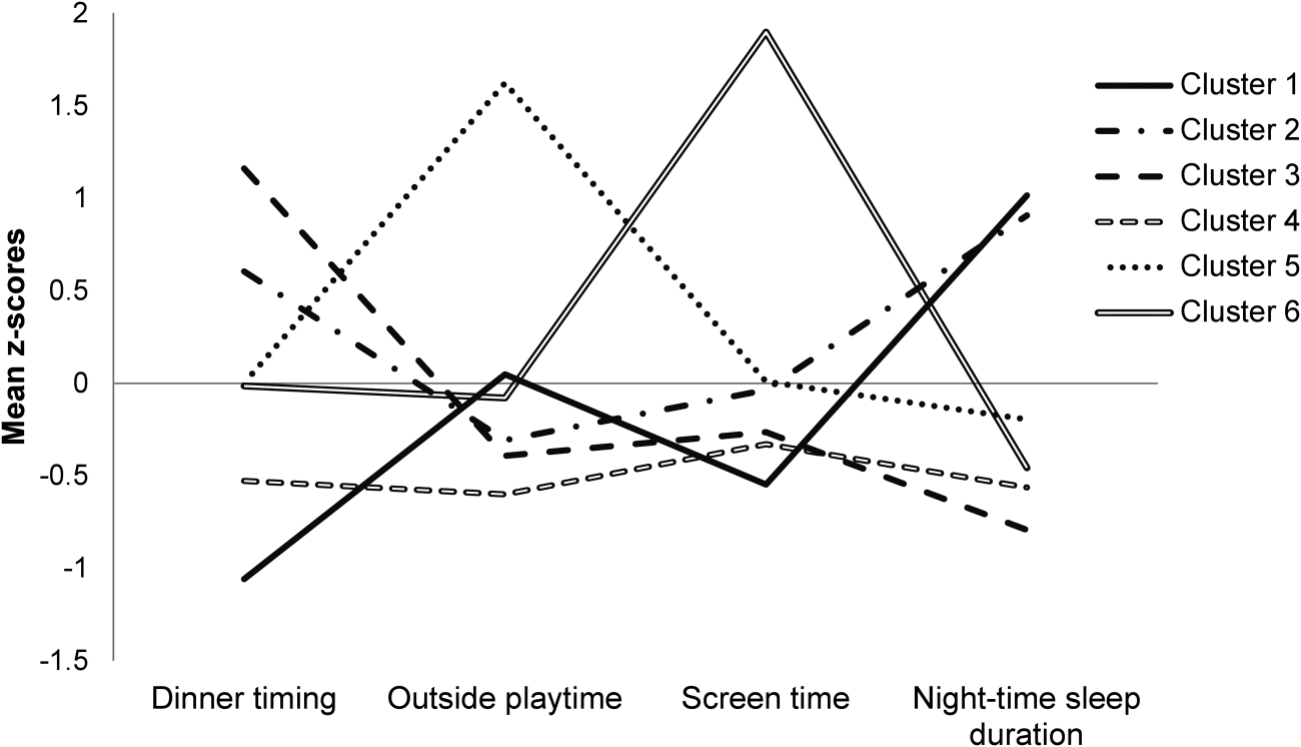
Final cluster centres (mean z-scores) of obesity-related lifestyle behaviours.

Six distinct clusters were identified. Characteristics of each cluster indicated by z-scores of lifestyle behaviours are shown in figure 1 and the raw mean values are shown in table 1. Cluster 1 (C1) was characterised by the earliest dinner timing, the least screen time and the longest night-time sleep duration. Cluster 2 (C2) had as much sleep duration as in C1, but the dinner timing was relatively late when compared with other clusters. Cluster 3 (C3) was characterised by late dinner timing and the shortest sleep duration. Cluster 4 (C4) had the least amount of outside playtime, whereas cluster 5 (C5) had the most outside playtime. Cluster 6 (C6) was characterised by having the most screen time and shorter sleep duration.

**Table 1:** Mean values of four obesity-related lifestyle behaviours by cluster pattern

	Cluster 1	Cluster 2	Cluster 3	Cluster 4	Cluster 5	Cluster 6	p Value comparing 6 clusters*	Adjusted significance level (rank)†
	n=268	n=271	n=257	n=336	n=238	n=175		
	Mean (SD)	Mean (SD)	Mean (SD)	Mean (SD)	Mean (SD)	Mean (SD)		
Dinner timing (pm)	5:57 (0:19)	6:48 (0:19)	7:05 (0:20)	6:13 (0:17)	6:30 (0:23)	6:29 (0:26)	<0.001	0.013 (1)‡
Outside playtime (hours/day)	1.7 (0.6)	1.4 (0.6)	1.3 (0.6)	1.2 (0.5)	3.1 (0.6)	1.6 (0.7)	<0.001	0.017 (2)‡
Screen time (hours/day)	1.5 (0.8)	2.1 (0.8)	1.8 (0.8)	1.8 (0.7)	2.1 (0.8)	4.2 (0.9)	<0.001	0.050 (4)‡
Night-time sleep duration (hours/day)	10.4 (0.4)	10.3 (0.4)	9.2 (0.4)	9.4 (0.4)	9.6 (0.5)	9.4 (0.5)	<0.001	0.025 (3)‡

*p Values calculated from analysis of variance (ANOVA).

†Adjusted significance level using the Holm's method^36^ for multiple testing, the first entry being the adjusted significance level and the rank in parentheses being the rank of the associated original p value in ascending order from most-to-least significant.

‡Statistically significant (p<0.05) after adjustment for multiple tests using the Holm's method.

The characteristics of participants by cluster pattern are shown in table 2. C1 and C2 had higher proportions of girls, whereas C4 and C5 consisted of more boys. Children's mean age was the highest in C5. However, all these characteristics of children and parents were not significantly different across clusters.

**Table 2:** Differences in characteristics of participants by cluster pattern

	Cluster 1	Cluster 2	Cluster 3	Cluster 4	Cluster 5	Cluster 6	p Value comparing 6 clusters	Adjusted significance level (rank)*†
	n=268	n=271	n=257	n=336	n=238	n=175		
*Children*								
Sex (%)								
Boys	47.0	49.0	54.5	58.6	58.4	53.1	0.017‡	0.007 (1)
Girls	53.0	51.0	45.5	41.4	41.6	46.9		
Age (years)	4.2 (0.9)	4.2 (0.8)	4.2 (0.8)	4.2 (0.9)	4.4 (0.9)	4.2 (0.9)	0.022§	0.008 (2)
3 years (%)	24.3	23.2	24.9	27.1	17.7	24.6	0.039‡	0.010 (3)
4 years (%)	38.4	34.3	38.1	28.0	30.2	34.3		
5 years (%)	32.5	38.8	33.5	40.2	45.4	34.3		
6 years (%)	4.8	3.7	3.5	4.7	6.7	6.8		
*Parents*								
Age (years)								
Mothers	33.3 (4.1)	34.0 (4.4)	33.7 (4.8)	33.6 (4.3)	33.0 (4.1)	33.0 (4.8)	0.049§	0.013 (4)
Fathers	36.0 (5.4)	36.3 (5.5)	36.2 (5.6)	36.4 (5.5)	35.5 (5.4)	36.0 (6.5)	0.592§	0.050 (7)
Overweight¶ (%)								
Mothers	7.5	5.8	9.8	8.2	5.6	12.3	0.143‡	0.017 (5)
Fathers	24.1	26.4	30.5	30.3	23.0	21.2	0.160‡	0.025 (6)

Values are provided as proportion or mean (SD).

*Adjusted significance level using the Holm's method^36^ for multiple testing, the first entry being the adjusted significance level and the rank in parentheses being the rank of the associated original p value in ascending order from most-to-least significant.

†All variables were not statistically significant after adjustment for multiple testing using the Holm's method.

‡p Values calculated from χ^2^ test.

§p Values calculated from analysis of variance (ANOVA).

¶Parents' overweight (including obese) defined as body mass index ≥25 kg/m^2^.^33^ Missing number of cases: mothers living with children (n=1532): mothers' age (65) and obesity (142); fathers living with children (n=1412): fathers' age (36) and obesity (88).

### Differences in children's weight status and family environments by cluster pattern

The prevalence of overweight in children was significantly different across clusters and was the lowest in C1 (4.0%) and the highest in C6 (15.1%) (table 3).

**Table 3:** Differences in children's overweight and family environments by cluster pattern

	Cluster 1	Cluster 2	Cluster 3	Cluster 4	Cluster 5	Cluster 6	p Value comparing 6 clusters*	Adjusted significance level (rank)†
	n=268	n=271	n=257	n=336	n=238	n=175		
*Children’s weight status*								
Non-overweight	96.0	93.2	91.6	91.0	89.0	84.9	0.007	0.010 (6)‡
Overweight§	4.0	6.8	8.4	9.0	11.0	15.1		
*Family environments*								
Family members living with children								
Parental status								
Two parents	90.6	95.2	90.7	89.9	91.2	86.9	0.079	0.013 (7)
One parent	9.4	4.8	9.3	10.1	8.8	13.1		
Presence of siblings								
None (only child)	20.1	19.2	23.0	21.4	16.4	26.3	0.199	0.017 (8)
One or more	79.9	80.8	77.0	78.6	83.6	73.7		
Presence of grandparents								
None	39.9	51.7	54.9	42.9	41.6	44.6	0.002	0.007 (4)‡
One or more	60.1	48.3	45.1	57.1	58.4	55.4		
Maternal employment status								
Unemployed	38.7	39.0	17.8	16.7	23.5	23.2	<0.001	0.006 (3)‡
Employed	61.3	61.0	82.2	83.3	76.5	76.8		
Habitual family and parents’ behaviours								
Meal regularity								
Regular	72.1	66.4	58.3	64.9	64.2	52.3	0.002	0.008 (5)‡
Irregular	27.9	33.6	41.7	35.1	35.8	47.7		
Habitual exercise (minutes/week)								
Mother								
<150	98.4	99.2	97.6	98.1	96.0	97.6	0.240	0.025 (9)
≥150	1.6	0.8	2.4	1.9	4.0	2.4		
Father								
<150	92.2	90.7	89.8	90.5	91.7	91.9	0.943	0.050 (10)
≥150	7.8	9.3	10.2	9.5	8.3	8.1		
Screen time (hours/day)								
Mother								
<2	54.0	48.3	53.0	52.7	40.5	20.0	<0.001	0.005 (1)‡
2–3	39.2	40.6	40.2	38.2	42.8	42.6		
≥4	6.8	11.1	6.8	9.1	16.7	37.4		
Father								
<2	46.8	37.4	37.9	42.5	30.3	15.4	<0.001	0.006 (2)‡
2–3	44.0	55.3	53.9	49.5	59.0	51.0		
≥4	9.2	7.3	8.2	8.0	10.7	35.6		

Values are provided as proportion.

*p Values calculated from χ^2^ test.

†Adjusted significance level using the Holm's method^36^ for multiple testing, the first entry being the adjusted significance level and the rank in parentheses the rank of the associated original p value in ascending order from most-to-least significant.

‡Statistically significant (p<0.05) after adjustment for multiple testing using the Holm's method. Missing number of cases: children's overweight (252), parental status (3), and meal regularity (286); mothers living with children (n=1532): maternal employment status (66), habitual exercise (65), and screen time (152); fathers living with children (n=1412): habitual exercise (58) and screen time (94).

§Children's overweight (including obese) defined as sex-specific and age-specific body mass index cut-off points of the International Obesity Task Force.^29^

For family members living with children, presence of grandparents and maternal employment status were significantly different across clusters. Living with one or more grandparents was a higher in C1 (characterised by the early dinner timing, the least screen time and the longest sleep duration), C4 (characterised by the least outside playtime) and C5 (characterised by the most outside playtime) and a lower proportion in C3 (characterised by late dinner time and the shortest sleep duration) and C2 (characterised by later dinner timing and longer sleep duration) across clusters. The proportion of employed mothers was lower in C1 and C2 and higher in C3 and C4. Neither parental status nor presence of siblings was significantly different across clusters.

For habitual family and parent behaviours, meal regularity and screen time in both parents were significantly different across clusters, although no differences were found for habitual exercise in either parent. The proportion of irregular meals was the lowest in C1 and the highest in C6 (characterised by the most screen time and shorter sleep duration). Marked differences were seen in parents' screen time. The proportion of excessive time spent in screen-viewing (≥4 hours/day) was highest in C6 compared with all other clusters for both parents.

## Discussion

This study examined preschool children's lifestyle behaviour clustering patterns (including dinner timing, outside playtime, screen time and night-time sleep duration) and their associations with children's overweight (including obese) and family environments. Cluster analysis identified six clusters, and the prevalence of being overweight varied across clusters, ranging from 4.0% to 15.1%. Family environments including irregular mealtimes and parents' excessive screen time differed among clusters.

The lifestyle behaviour pattern with the highest risk of being overweight (C6) had the most screen time, shorter sleep duration and average dinner timing and outside playtime compared with the other clusters. Those with the lowest risk of being overweight (C1) had the least screen time, the longest sleep duration, the earliest dinner timing and average outside playtime. Focusing on screen time and night-time sleep duration, in which notable differences were observed among the clusters, the patterns with either less screen time or longer sleep duration (C2: average screen time and long sleep duration, C3: less screen time and short sleep duration) and those with both (C1) showed lower risk of overweight than the cluster with neither behaviours (C6), regardless of dinner timing and outside playtime. These results are supported by other studies demonstrating that more screen time and short sleep duration were independent risk behaviours for childhood overweight.^1^ ^3^ ^5^ ^7^ ^9^ ^10^ In addition, a negative association between screen time and sleep duration has been found^3^ and increased screen time may lead to further decrease in sleep duration. This suggests, therefore, that decreased screen time and increased sleep duration could be important behaviours for achieving or maintaining a healthy body weight status in children.

The lifestyle behaviour pattern with the highest risk of overweight was associated with a family environment having more screen time for both parents, not just children. These findings are consistent in showing that a high frequency of parents who spent more screen time was associated with children's increased screen time.^26^ ^27^ Stamatakis *et al*^37^ has reported that excessive screen time in adults is associated with increased mortality and cardiovascular disease risk regardless of physical activity participation, which demonstrates that decreased screen time is a favourable behaviour in parents as well as in children.

Children with the lifestyle behaviour pattern having the highest risk of overweight were also in family environments having a substantially higher proportion of irregular mealtimes as a family, although dinner timing was average. In contrast, the lifestyle behaviour pattern having the lowest risk of overweight was in family environments with the lowest proportion of irregular mealtime and the earliest dinner timing across clusters. These results suggest that mealtime regularity may be more important than dinner timing for children's overweight. Although no studies were identified that examined the association between irregular mealtimes and other lifestyle behaviours, having irregular mealtimes may provide children more opportunity for watching TV while waiting for a meal and could lead to increased screen time and decreased night-time sleep duration. A public health approach should focus on modifying these family environments to achieve and promote healthy lifestyle behaviour patterns in children along with their parents.

For family members, children in the clusters with a higher proportion of employed mothers (C3, C4, C5 and C6) had lifestyle behaviour patterns with shorter sleep duration and higher prevalence of overweight than the other two clusters. These findings are consistent with studies showing that the length of mothers' working hours was negatively associated with children's sleep duration^22^ and that maternal employment was associated with children's overweight.^30^ ^31^ Our previous study found that living with grandparents was more likely to contribute to children's overweight than maternal employment.^32^ In the current study, children in the clusters with a higher proportion of living with grandparents (C4, C5 and C6) had also a higher prevalence of overweight than those with a lower proportion (C3), except the two clusters with a lower proportion of employed mothers. In contrast, children from clusters in which a higher proportion lived with at least one grandparent (C1, C4, C5 and C6) had lifestyle behaviour patterns with early dinner timing than the children in the other two clusters. Although there is no study that has examined an association between mealtimes and the presence of grandparents, it is considered that grandparents who live with children may play supportive roles in caring for children and/or in preparing meals for the children and the family. Thus, maternal employment and presence of grandparents are environmental factors that influence children's habitual behaviours such as sleep duration and dinner timing, and lifestyle behaviour patterns combined with these behaviours influence children's body weight status.

Among four lifestyle behaviours we examined, dinner timing and outside playtime were not consistently associated with children's overweight. Dinner timing is a behaviour that affects skipping breakfast^38^ and skipping breakfast is associated with children’s overweight.^39^ Thus, dinner timing is considered as an important dietary behaviour. Although an association between late dinner timing (after 20:00) and high BMI has been reported in adults,^6^ no studies have examined this in children. Our results could not determine whether the mealtime was early or late enough to affect children's overweight. For outside playtime, the average time was 1.2 hours/day in the shortest cluster and exceeded the physical activity recommendation for children in all clusters (60 min/day).^28^ Although the current study did not examine the intensity of children's activity, a study that assessed preschool children's physical activity in direct observation has reported that time spent outdoors was positively associated with physical activity.^40^ Thus, it is possible that these children had a sufficient active level of physical activity because they spent much time outdoors.

The present study has several limitations. First, this study was a cross-sectional design and therefore a causal relationship cannot be identified. Second, measurements were based on the principal caregivers' reports, although children's weight and height were measured at each childcare facility. Principal caregivers who directly observe children's daily behaviours were asked to report children's behaviours. Also, all behaviour time variables were separately constructed on weekdays and on weekend days in order to increase accuracy. However, proxy-reporting may have introduced recall and social desirability bias.^41–43^ Further research is needed to explore comprehensive lifestyle behaviour patterns used in objective measurements. Third, socioeconomic status, such as parents' educational level and/or household economic level, might affect the children's overweight and behaviours, but our study could not include these kinds of variables. Fourth, the data used in our study were collected in 2003, thus lifestyle behaviours may not necessarily reflect frequencies and proportions of recent lifestyle behaviours. However, the influences of lifestyle behaviour patterns on body weight status and family environments on children's lifestyle behaviour patterns can be considered to be unchanged over time. Despite these limitations, the current study surveyed almost the same city as the average household income in Japan.^44^ The survey was conducted on all children attending childcare facilities in a city with more than 100 000 in population and included 93.3% of the children living in that area and yielded a relatively high response rate (73.1%). Thus, our study covered a wide range of preschool-aged children's lifestyle behavioural characteristics.

In conclusion, this study found that the children's lifestyle behaviour pattern (characterised by more screen time, short sleep duration and average dinner timing and outside playtime) is associated with the highest risk of overweight and is shaped by family environments with irregular mealtimes and more screen time in both parents. The study findings emphasise a public health approach to shape children's healthy lifestyle behaviour patterns, especially decreasing screen time and increasing night-time sleep duration, should focus on family members living with children, as well as on children, and should focus on modifying family environments, such as having regular mealtimes as a family and decreasing parents' screen time.
